# Changes in Adult Obesity Trends in the US

**DOI:** 10.1001/jamahealthforum.2024.3685

**Published:** 2024-12-13

**Authors:** Benjamin Rader, Rebecca Hazan, John S. Brownstein

**Affiliations:** 1 Computational Epidemiology Lab, Boston Children’s Hospital, Boston, Massachusetts; 2 Department of Anaesthesia, Harvard Medical School, Boston, Massachusetts; 3 Optum Life Sciences, Boston, Massachusetts; 4 Department of Pediatrics, Havard Medical School, Boston, Massachusetts

## Abstract

This cross-sectional study examines US trends in adult obesity prevalence from 2013 to 2023.

## Introduction

The prevalence of obesity in the US has increased for several decades, including during the COVID-19 pandemic.^1,2^ Some long-term forecasts estimate this upward trajectory will continue, while others forecast a plateau.^3,4^ As the US transitions from pandemic conditions and weight loss medication use (eg, semaglutide) becomes more common, near-term changes in obesity prevalence are unclear. We examined national trends in body mass index (BMI) and obesity among US adults (aged ≥18 years) between January 1, 2013, and December 31, 2023.

## Methods

This cross-sectional study used the Optum deidentified Market Clarity Data of linked medical and insurance claims^5^ and electronic health records.^6^ The study was a secondary use of fully deidentified data; thus, local ethics review and informed consent were not required in accordance with the Common Rule. The study followed the STROBE reporting guideline.

All US medical groups continuously contributing data from 2013 through 2023 were included. US adults with electronic health record data were included regardless of patient insurance status or presence of claims data. For nonpregnant adults, we extracted highest BMI measurement (weight in kilograms divided by height in meters squared) recorded each calendar year and select demographic variables associated with BMI, including self-reported race and ethnicity (Asian, Black, Hispanic, White, and other or unknown), age, and geographic region from all outpatient visits.

Mean population BMI and percentage of adults with obesity (BMI ≥30) were calculated annually and by demographic strata. Measures were weighted for demographic variables to target 2020 census estimates. Obesity estimates were compared with World Health Organization (WHO) measurements (2013-2015) and projections (2016-2022). A sensitivity analysis of unweighted data was also performed. In a subset of insured patients with available claims data in 2023, the unadjusted percentage of patients with glucagon-like peptide 1 receptor agonist (GLP-1RA) dispensing claims were analyzed by geographic region. Further details are included in the eMethods in Supplement 1. Data analyses were performed using R, version 4.3.2 (R Foundation).

## Results

A total of 16 743 822 unique adults (78.4% aged 26-75 years; 51.3% female and 48.7% male) (Table) contributed 47 939 382 BMI measurements. Mean (SD) population BMI rose annually from 2013 (29.65 [1.99]) to 2021 (30.23 [2.04]), plateaued in 2022 (30.24 [2.04]), and decreased slightly in 2023 (30.21 [1.99]). This same pattern was seen in percent changes of adults with obesity (Figure, A). While point estimates differed, obesity trends in the Market Clarity Data mirrored WHO trends until 2021.

**Table.** Demographics of 16 743 822 US Adults Contributing Body Mass Index Measurements From the Optum Deidentified Market Clarity Data

Characteristic	%											
	2013	2014	2015	2016	2017	2018	2019	2020	2021	2022	2023	US Census–weighted targets^a^
Region												
Midwest	45.3	44.3	44.5	48.8	47.8	44	42.5	41.2	40.5	38.4	36.4	20.8
Northeast	13.6	13.5	13.2	11.8	12.1	13.1	13.6	14.3	14.4	14.9	15.7	17.5
South	32.2	32.8	32.7	30.3	30.7	33.5	34.2	34.8	34.8	36.0	37.2	37.9
West	8.9	9.4	9.5	9.1	9.4	9.3	9.7	9.7	10.3	10.7	10.7	23.7
Age group, y												
18-25	9.9	9.8	9.6	9.3	9.1	9	8.9	8.6	8.7	8.6	8.4	13.9
26-35	12.6	12.5	12.2	11.9	11.7	11.6	11.4	11.1	11.2	11.1	11.4	17.9
36-45	14.8	14.4	14.2	14.0	13.6	13.6	13.5	13.2	13.4	13.5	13.7	16.2
46-55	19.1	18.5	18.2	18.0	17.6	17.1	16.6	16.2	15.9	15.7	15.6	16.5
56-65	18.9	19.1	19.4	19.7	19.9	19.9	19.9	20.0	19.7	19.3	18.8	16.4
66-75	13.9	14.5	15	15.5	16.2	16.7	17.1	17.9	18.1	18.5	18.4	11.4
≥76	10.8	11.2	11.4	11.6	12.0	12.2	12.5	13.0	12.9	13.3	13.7	7.8
Sex												
Female	58.5	58.1	57.8	57.4	57.1	57.1	57.2	57.1	57.2	57.4	58.4	51.3
Male	41.5	41.9	42.2	42.6	42.9	42.9	42.8	42.9	42.8	42.6	41.6	48.7
Race												
Asian	2.1	2.1	2.3	2.3	2.4	2.5	2.6	2.5	2.8	2.9	3.1	5.8
Black	12.8	13.1	13.2	12.5	12.6	13.3	13.1	13.2	13.2	13.3	13.0	12.3
White	80.6	80.1	79.9	80.3	80.0	79.2	79.2	78.9	78.3	77.6	77.2	72.2
Other^b^ or unknown	4.5	4.7	4.6	4.9	5.0	5.1	5.2	5.4	5.8	6.2	6.6	9.7
Ethnicity												
Hispanic	5.6	5.8	6.0	6.1	6.2	6.4	6.8	7.2	7.7	8.3	9.1	16.1
Non-Hispanic	94.4	94.2	94.0	93.9	93.8	93.6	93.2	92.8	92.3	91.7	90.9	83.9

^a^ Coarse demographic groups were used and weights were not raked or trimmed; therefore, when weighting was applied, the target population demographics were successfully approximated each year to within <0.1% of each subgroup.

^b^ Other self-reported races were collapsed to preserve anonymity.

**Figure.**
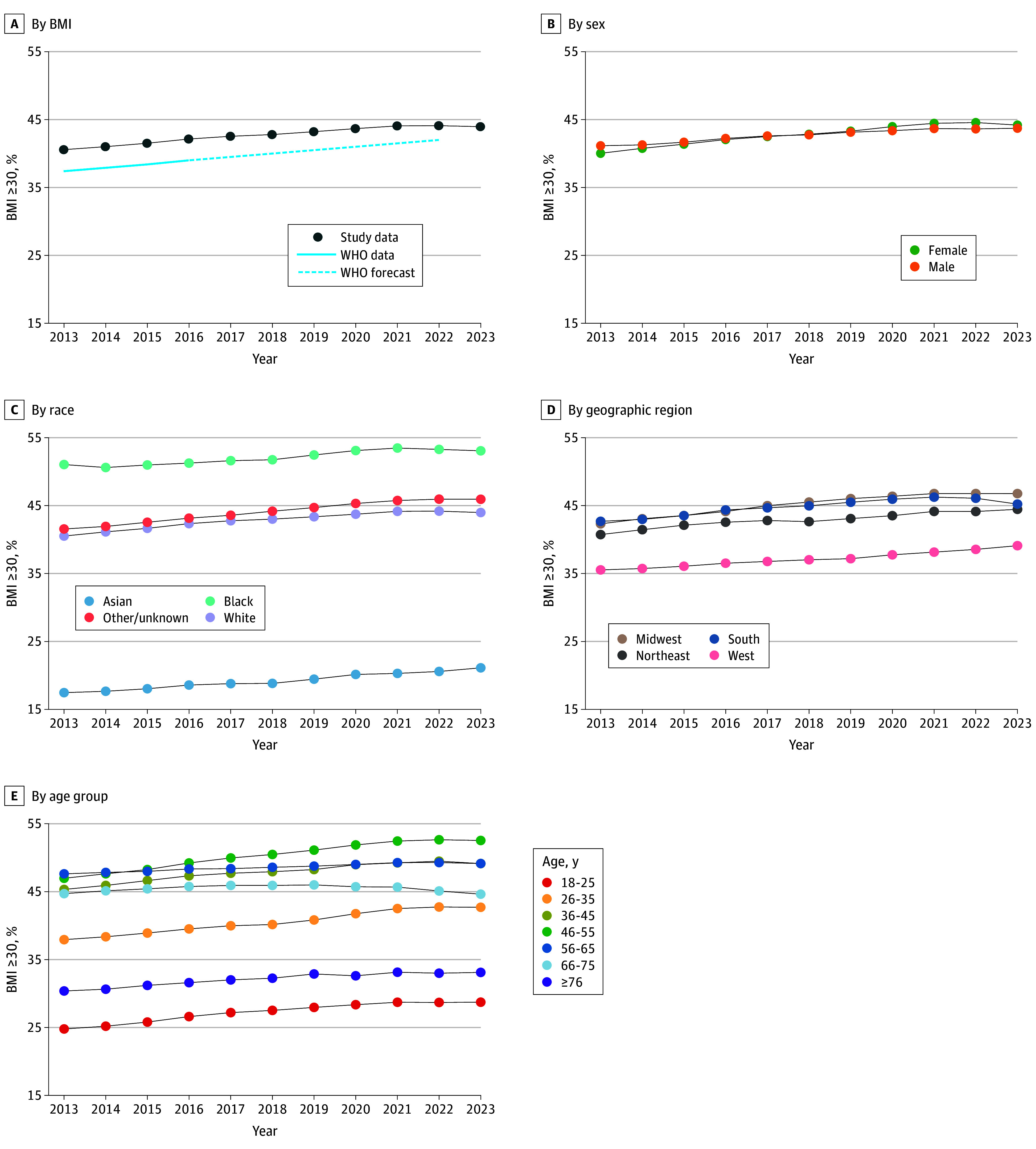
US Adult Population With Obesity Weighted by Sex, Race, Geography, and Age to Target US Census Estimates Percentage of US adults with obesity was calculated annually and by demographic strata in 16 743 822 unique patients from the Optum deidentified Market Clarity Data of linked medical and insurance claims and electronic health records between January 1, 2013, and December 31, 2023. BMI indicates body mass index (calculated as weight in kilograms divided by height in meters squared); WHO, World Health Organization.

A decrease in obesity prevalence was observed in the South, among individuals aged 66 to 75 years, and among females (Figure, B-E). The 2023 decline in obesity prevalence was also seen in a sensitivity analysis using unweighted data (46.2%, 46.0%, and 45.6% in 2021, 2022, and 2023, respectively). For a subset of 10 625 745 individuals with available 2023 insurance claims, GLP-1RA dispensing differed by region (South, 6.0%; Midwest, 5.1%; Northeast, 4.4%; West, 3.4%).

## Discussion

These findings suggest that BMI and obesity prevalence in the US decreased in 2023 for the first time in more than a decade. The most notable decrease was in the South, which had the highest observed per capita GLP-1RA dispensing rate. However, dispensing does not necessarily mean uptake, and the South also experienced disproportionately high COVID-19 mortality among individuals with obesity.^2^

Obesity and BMI are imperfect proxies for adiposity; thus, future studies should investigate alternative body composition measures and potential causes for the observed shifts, including GLP-1RA proliferation (eg, out-of-pocket purchases) or pandemic-associated demographic and behavior changes. This study is limited by possible selection bias and compositional changes, as BMI recorded during medical visits may have skewed estimates and may explain the slightly higher obesity prevalence in this dataset. However, early trends mirrored WHO authoritative data. Statistical tests were omitted to prevent interpretation of small *P* values from large samples as evidence of no systemic bias. While obesity remains a considerable public health concern, the observed reductions in obesity prevalence suggest an encouraging reversal from long-standing prior increases.

## Notes

Supplement 1.
eMethods

Supplement 2.
Data Sharing Statement
