# Supplementary material for: Changes in Adult Obesity Trends in the US
Source: JAMA Health Forum. 2024 Dec 13;5(12):e243685. doi: 10.1001/jamahealthforum.2024.3685 (PMC11645646; doi:10.1001/jamahealthforum.2024.3685)
Supplement: Supplement 1. — eMethods [file jamahealthforum-e243685-s001.pdf]

Supplemental Online Content

Rader B, Hazan R, Brownstein JS. Changes in adult obesity trends in the US. *JAMA Health Forum*. 2024;7(11):e243685. doi:10.1001/jamahealthforum.2024.3685

eMethods

This supplemental material has been provided by the authors to give readers additional information about their work.

© 2024 Rader B et al. *JAMA Health Forum*.

eMethods

Dataset

Optum's de-identified Market Clarity Data (Market Clarity) is an integrated, multi-source medical claims, pharmacy claims, and electronic health records data set. Market Clarity links electronic health record data - including demographics, lab results, vital signs and measurements, diagnoses, procedures and information derived from unstructured clinical notes using natural language processing - with historical, linked administrative claim data - including pharmacy claims, physician claims, clinical information facility claims and medications prescribed and administered. All patients in Market Clarity have electronic health records data. Patients come from any Optum-partnered provider and therefore Market Clarity is comprised by patient visits with >150 payers including commercial insurance, Medicaid, Medicare, and uninsured individuals. A subset of patients have linked claims data. This subset does not include uninsured individuals.

Variables

**Variable**	**Method**	**Level**	**Source**
Body mass index	Calculated from provider measured height and weight	Visit	Electronic health record
Age	Calculated from self-reported year of birth	Visit	Electronic health record
U.S. Census Region	Derived from self-reported home address	Patient	Electronic health record
Race	Aggregated from self-reported race	Patient	Electronic health record
Ethnicity	Aggregated from self-reported ethnicity	Patient	Electronic health record
GLP-1 receptor agonist dispensing	Insurance claim for GLP-1 receptor agonist*	Claim	Claims data linked to patient's electronic health record

Weighting

We calculated post-stratification weights based on participants' self-reported characteristics, including age, sex, race (Black, White, Asian, Other/Unknown), ethnicity (Hispanic vs. Non-Hispanic), and geography (U.S. Census Region, derived from home address). Note, coarse demographic categories and "other/unknown" race were used to preserve patient anonymity and prevent patient reidentification.

These weights were designed to target the joint distribution of these characteristics as reported in the 2020 U.S. Census Public Use Microdata Sample (PUMS) data. Note that by generating weights to standardize each year to the 2020 U.S. population, our sample is intended to represent a consistent demographic composition across all years of the study. Because we utilized coarse demographics groups, and did not rake or trim weights, we were able to successfully weight to the target population each year. This approach allowed us to assess changes in obesity trends within a stable population framework, independent of demographic shifts over time. However, it also means that the reported weighted outcomes do not account for any changes in the U.S. population's demographic makeup during the study period.

© 2024 Rader B et al. *JAMA Health Forum*.

**GLP-1 Receptor Agonists*
A 2023 dispensing off a GLP-1 receptor agonist was defined as an insurance claim for any of the following: dulaglutide, exenatide, liraglutide, lixisenatide, semaglutide, tirzepatide, and exenatide microspheres.

World Health Organization Comparison Data
World Health Organization data on age-standardized prevalence of obesity among adults (18+ years) is available: https://data.who.int/indicators/i/C6262EC/BEFA58B

© 2024 Rader B et al. *JAMA Health Forum*.
