# Supplementary material for: Changes in Adult Obesity Trends in the US
Source: JAMA Health Forum. 2024 Dec 13;5(12):e243685. doi: 10.1001/jamahealthforum.2024.3685 (PMC11645646; doi:10.1001/jamahealthforum.2024.3685)
Supplement: Supplement 2. — Data Sharing Statement [file jamahealthforum-e243685-s002.pdf]

Data Sharing Statement

Rader. Changes in Adult Obesity Trends in the US. *JAMA Health Forum*. Published December 13, 2024. doi:10.1001/jamahealthforum.2024.3685

Data
Data available: No

Additional Information
Explanation for why data not available: Raw data are not publicly available and can be licensed for use from Optum. Aggregate data is available by request to the corresponding author.
